# Galectin-4 Controls Intestinal Inflammation by Selective Regulation of Peripheral and Mucosal T Cell Apoptosis and Cell Cycle

**DOI:** 10.1371/journal.pone.0002629

**Published:** 2008-07-09

**Authors:** Daniela Paclik, Silvio Danese, Uta Berndt, Bertram Wiedenmann, Axel Dignass, Andreas Sturm

**Affiliations:** 1 Medizinische Klinik m.S. Hepatologie und Gastroenterologie, Campus Virchow Klinikum, Charité-Universitätsmedizin Berlin, Berlin, Germany; 2 Division of Gastroenterology, Istituto Clinico Humanitas-IRCCS, Milan, Italy; 3 Medizinische Klinik I, Markus Krankenhaus, Frankfurt, Germany; The Rockefeller University, United States of America

## Abstract

Galectin-4 is a carbohydrate-binding protein belonging to the galectin family. Here we provide novel evidence that galectin-4 is selectively expressed and secreted by intestinal epithelial cells and binds potently to activated peripheral and mucosal lamina propria T-cells at the CD3 epitope. The carbohydrate-dependent binding of galectin-4 at the CD3 epitope is fully functional and inhibited T cell activation, cycling and expansion. Galectin-4 induced apoptosis of activated peripheral and mucosal lamina propria T cells via calpain-, but not caspase-dependent, pathways. Providing further evidence for its important role in regulating T cell function, galectin-4 blockade by antisense oligonucleotides reduced TNF-alpha inhibitor induced T cell death. Furthermore, in T cells, galectin-4 reduced pro-inflammatory cytokine secretion including IL-17. In a model of experimental colitis, galectin-4 ameliorated mucosal inflammation, induced apoptosis of mucosal T-cells and decreased the secretion of pro-inflammatory cytokines. Our results show that galectin-4 plays a unique role in the intestine and assign a novel role of this protein in controlling intestinal inflammation by a selective induction of T cell apoptosis and cell cycle restriction. Conclusively, after defining its biological role, we propose Galectin-4 is a novel anti-inflammatory agent that could be therapeutically effective in diseases with a disturbed T cell expansion and apoptosis such as inflammatory bowel disease.

## Introduction

Although the pathogenesis of inflammatory bowel diseases (IBD) is not fully understood, it is evident that both Crohn's disease (CD) and ulcerative colitis (UC) are linked to a failure of the mucosal immune system and that both an unrestricted expansion and an impaired apoptosis of T cells foster mucosal inflammation [1], [2]. Thus, therapeutic approaches inhibiting T cell proliferation such as steroids, azathioprine/6-MP or calcineurin inhibitors or drugs inducing T cell apoptosis such as tumor necrosis factor-α antibodies are effectively used to treat CD and UC [3], [4].

Galectins are a family of animal lectins defined by their recognition of β-galactose and the presence of consensus amino acid sequences [5]. They exhibit high levels of evolutionary conservation in their carbohydrate-recognition domains (CRDs) of about 130 amino acids responsible for carbohydrate binding. With respect to the gastrointestinal (GI) tract, galectin-4 (Gal-4) is of specific importance since RNAse protection assays have demonstrated that it is expressed in gastrointestinal tissues, but not in brain, kidney, skeletal muscle, heart, liver or lung tissue [6].

Since in human inflammatory disorders the ultimate goal is to become inflammation-free, it will therefore be necessary to find a therapeutic approach which not only blocks one or more components of specific pro-inflammatory pathways, such as T cell expansion and cytokine release, but also restores naturally occurring anti-inflammatory systems, such as apoptosis, which may be defective either in expression level or function in IBD. Since galectins in general are able to inhibit inflammation and induce apoptosis, we performed a step-by-step analysis of the effect of gastrointestinal Gal-4 on human T cells responsible for triggering inflammation and investigated the therapeutic effect of Gal-4 in an animal model of experimental colitis.

## Results

### Expression of Gal-4 in T cells

**Figure 1:**
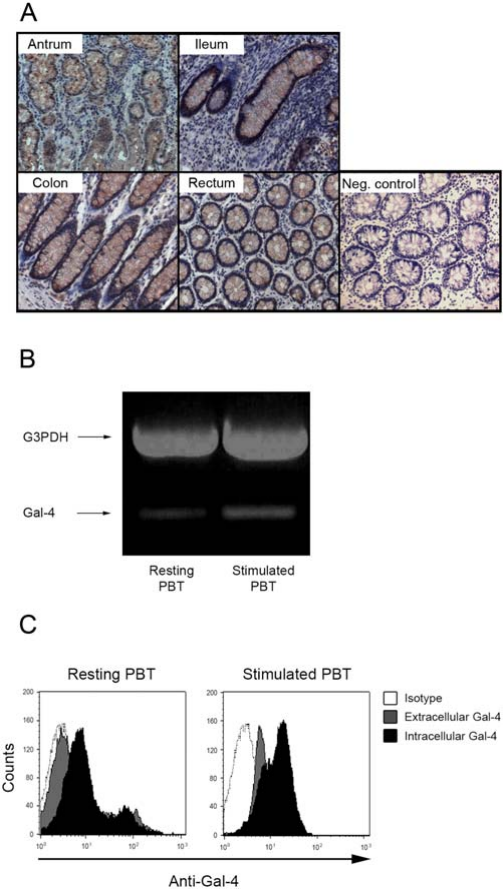
Identification of Galectin-4 expression. (A) Gal-4 expression was detected in cryosections of the GI tract of healthy volunteers. The results are representative for four individuals (original magnifications: ×100). (B) PCR analysis of Gal-4 mRNA expression in resting and anti-CD3/CD28 stimulated PBT. (C) Flow cytometric analysis of intra- and extracellular Gal-4 content in resting and anti-CD3/CD28 stimulated PBT. Data are representative of three individual experiments.

Whereas it is known that in the porcine and murine small intestine Gal-4 is expressed in epithelial cells [7], its expression pattern in the human GI tract remains unknown. When the entire human GI tract was sequentially characterized by immunohistochemical staining, strong Gal-4 staining was observed in epithelial cells of the antrum, ileum, colon and rectum (Fig. 1A) as well as in the corpus, fundus, and jejunum (not shown). In contrast, only weak Gal-4 positivity was detected in the underlying lamina propria cells (Fig. 1A) and absent in liver and spleen (not shown). Accordingly, Gal-4 mRNA expression in T cells was weak, but detectable (Fig. 1B) using GAPDH as a house-keeping gene. When the Gal-4 expression was quantified on the protein level, Gal-4 was detected intra- and extracellularly in PBMC, regardless of their activation status (Fig. 1C). Having defined that Gal-4 is strongly expressed in gastrointestinal epithelial cells, we next investigated, if Gal-4 is also secreted by epithelial cells and thus can interact with the underlying T cells in the lamina propria.

### Gal-4 secretion

To determine if Gal-4 is secreted by epithelial cells, HT-29, and Caco-2 cells were cultured for 48 h after which Gal-4 secretion was determined by a Gal-4 ELISA. Constantly, both cell lines secreted within this timeline about 45±6 pg/ml Gal-4. Having defined that Gal-4 is secreted by epithelial cells, we next addressed the question of whether Gal-4 binds to T cells, obligatory to modulate their function.

### Carbohydrate-dependent binding of Gal-4 to T cells

**Figure 2:**
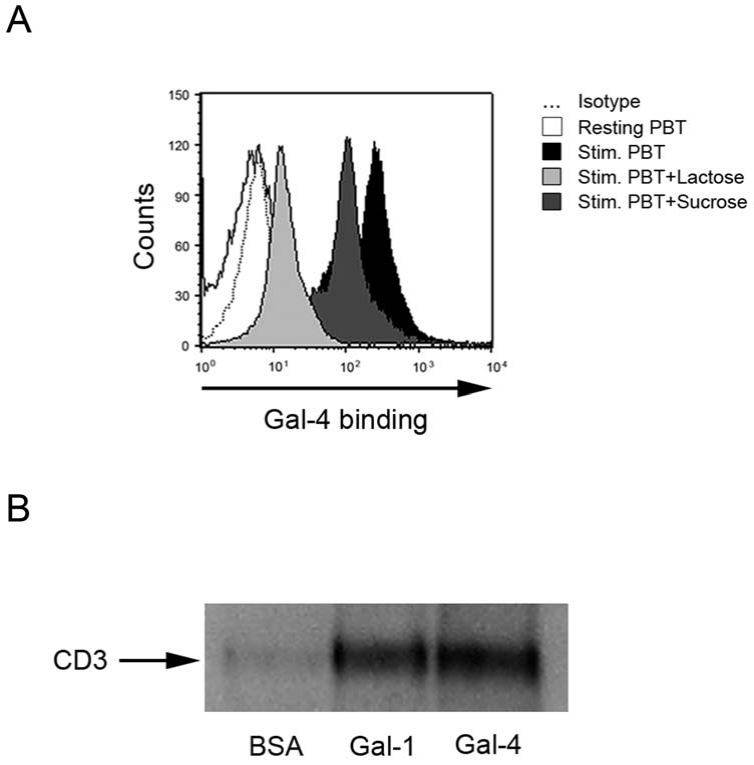
Galectin-4 binds to stimulated, but not resting T cells. (A) Flow cytometric analysis of Gal-4 binding to resting and anti-CD3/CD28 stimulated T cells using Gal-4 detected by an anti-Gal-4 Ab and PE-labeled secondary Ab. Carbohydrate-dependence of the binding was analysed by addition of 50 mM lactose as a pan-galectin inhibitor and 50 mM sucrose as control. Data are representative of three individual experiments. (B) Immunoprecipitation of Gal-4 binding complexes. BSA served as negative, Gal-1 as positive control. Data are representative for four individual experiments.

To investigate Gal-4 binding to T cells, Gal-4 was added to resting and stimulated peripheral blood and lamina propria (LPT) T cells and binding was quantified by flow cytometric analysis. Gal-4 did not bind to unstimulated, but to anti-CD3 and anti-CD2 stimulated PBT and LPT, respectively. This binding was β-galactoside-specifically, since the presence of 50 mM lactose as a pan-galectin inhibitor significantly reduced its binding (Fig. 2A). Sucrose tested at the same concentration failed to affect the staining intensity in flow cytometry, excluding non-specific effects (Fig. 2A). Next, we wanted to gain insight into the biochemical nature of cell surface targets for Gal-4.

### Gal-4 associates with CD3 but not with CD7 or β_1_-integrin

We and others previously described that CD3, CD7, and integrin-β_1_ have ligand capacity for Gal-1 and -2 [8], [9]. Prior work by Hokama et al. provided first evidence that Gal-4 interacts with the immunological synapse [10], however the respective binding region remained unclear. Thus, to scan the cell surface targets for Gal-4 on T cells and specify the exact binding side, we performed immunoprecipitation for Gal-4, followed by immunoblotting for CD3, CD7, and integrin-β_1_. Similar to Gal-1, but in contrast to Gal-2, CD3 was detected after affinity fractionation of extract with immobilized Gal-4 (Fig. 2B). In contrast to Gal-1 and -2, no trace of β_1_-integrin or CD7 was detected (not shown). A BSA control ascertained the absence of non-specific protein-protein interactions (Fig. 2B). Gal-1 served as a positive control (Fig. 2B), since it known that it bind at the CD3 receptor complex [9]. The CD3 receptor complex is required for cell-surface expression of the antigen-binding chains and signaling. Having demonstrated that Gal-4 homes in on CD3 or a tightly associated glycoprotein as major target, we hypothesized that Gal-4 modulates central T cell functions and went on to investigate the effect of Gal-4 on T cell activation and proliferation.

### Gal-4 inhibits T cell activation and proliferation

**Figure 3:**
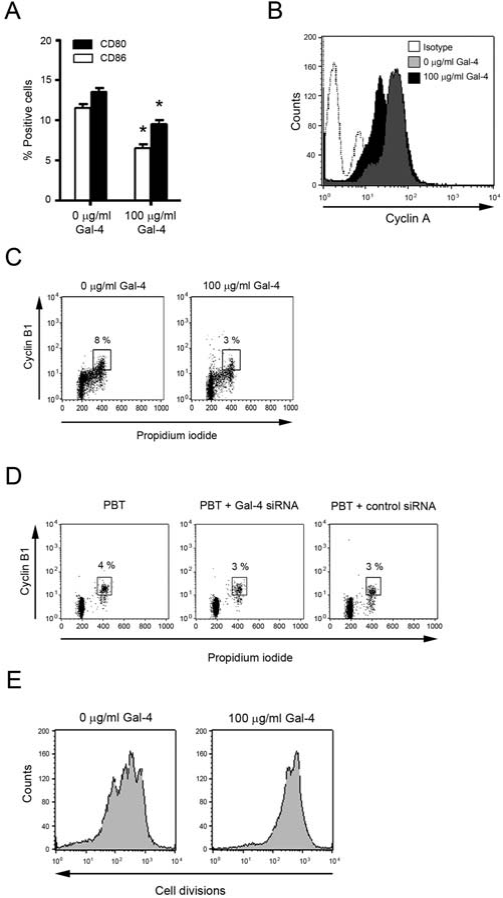
Galectin-4 inhibits activation, cell cycle progression, and expansion of activated T cells. (A) PBMC were stimulated with anti-CD3 and cultured for 72 h in the presence or absence of 100 µg/ml Gal-4. CD80 and CD86 positive cells were detected by flow cytometric analysis. Data represent mean±SEM of three individual experiments. *p≤0.05 for decrease vs. baseline. (B) Flow cytometric analysis of cyclin-A expression of anti-CD3/CD28 stimulated T cells cultured in the presence or absence of 100 µg/ml Gal-4. Data are representative for four individual experiments. (C) Flow cytometric analysis of cell cycle progression and cyclin-B1 expression of anti-CD3/CD28 stimulated T cells cultured in the presence or absence of 100 µg/ml Gal-4. Data are representative for four individual experiments. (D) PBT were transfected with Gal-4 siRNA or scrambled control. After stimulation with IL-2 cell cycle progression and cyclin B1 expression were determined. Data are representative of three individual experiments. (E) Anti-CD3/CD28 activated PBT were stained with CFDA and T cell expansion was determined after four days of incubation in the presence or absence of 100 µg/ml Gal-4 by flow cytometric analysis. Data are representative of three individual experiments.

Activation by costimulatory molecules is required to fully stimulate T cells and avoid anergy [11]. To determine T cell activation and proliferation, PBMC were activated with plate-bound CD3 mAb and incubated at the same time with or without 100 µg/ml Gal-4. Dead cells were excluded for further cell cycle analysis by proper gating on the respective DNA content. As demonstrated by flow cytometry analysis, Gal-4 significantly inhibited expression of the co-stimulatory molecules CD80 and CD86 expression on PBMC (Fig. 3A). Correspondingly, expression of IL-2 receptor-α chain (CD25), a sensitive marker for T cell activation and post-translational protein phosphorylation, determined by p-Tyr western blotting, were significantly down-regulated in Gal-4 co-cultured PBT (not shown). Of note, 100 µg/ml Gal-4 did not activate T cells in the absence of concurrent CD3 stimulation (not shown). T cell activation initiates cell cycling, a complexly regulated movement through different phases. Thus, we tested the effect of Gal-4 on PBT cell cycling separately for every cell cycle phase. The analysis of cyclin A, regulating the S-phase, and cyclin B1, propelling the cells from late G2 to mitosis, was measured in conjunction with propidium iodide (PI) staining to exactly localize its increase within the cell cycle and to exclude dead cells. The analysis revealed that Gal-4 potently inhibited T cell cycling (Fig. 3B and C). Additional BrdU-staining revealed that Gal-4 did not kill cells at a specific point in the cell cycle (not shown).

Interestingly, levels of key cell cycle inhibitors p21, p27, and p53 did not significantly differ in control and Gal-4 co-cultured activated T cells (not shown). Galectins are present intracellular, but can also be secreted [12] thus, we next aimed to compare the function of extracellular and intracellular Gal-4 on T cells. Therefore, we down-regulated Gal-4 expression with specific antisense oligonucleotides. As verified by TaqMan real time PCR, Gal-4 expression was inhibited by the specific antisense oligonucleotides by 49±7%. Interestingly, in contrast to extracellular Gal-4 addition, Gal-4-blockade had no impact on cyclin B1 expression (Fig. 3D).

The final goal of T cell cycling is to clonally expand the cell population necessary to provide a sufficient adaptive immune response. As determined by CFDA staining, after four days of stimulation with anti-CD3/CD28 mAb three T cell daughter populations were detected. In contrast, after equal stimulation of T cells in the presence of Gal-4 only one daughter cell population was detected (Fig. 3E). So far, we demonstrated on multiple levels, that Gal-4 potently inhibits T cell activation and consequently T cell cycling and expansion. We went on to determine the effect of Gal-4 on T cell cytokine secretion.

### Modulation of cytokine secretion in stimulated PBT and LPT by Gal-4

**Figure 4:**
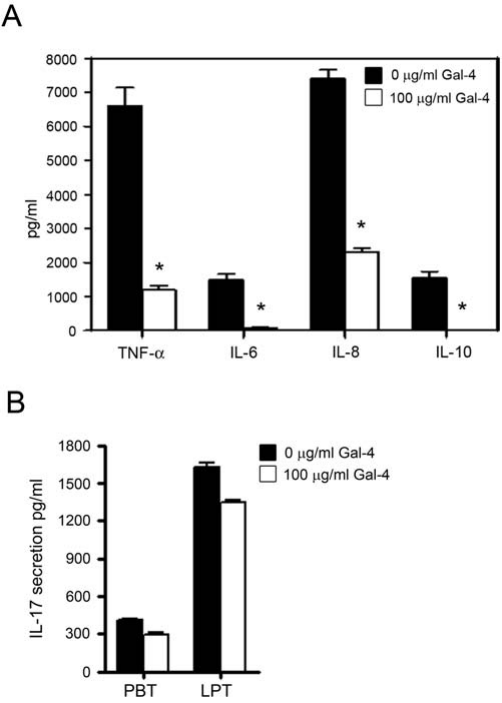
Galectin-4 and its domains distinctively reduce secretion of pro-inflammatory cytokines. (A) Cytokine release was tested in PBT stimulated with anti-CD3/CD28 for 72 hours. Data represent mean±SEM of six individual experiments. *p≤0.01 for decrease vs. baseline. (B) IL-17 secretion of LPT and PBT was determined by an IL-17 specific ELISA. LPT and PBT were activated by anti-CD2 or -CD3/CD28 mAb, respectively and cultured in the presence or absence of 100 µg/ml Gal-4. Data represent mean±SEM of three individual experiments.

The release of cytokines is crucial for the differentiation of T cells [13]. Having demonstrated that Gal-4 substantially inhibits PBT cell cycling, we were interested in whether Gal-4 modulate cytokine release patterns in T cells. PBT were isolated and stimulated with anti-CD3/CD28 mAb in the presence or absence of Gal-4 for 72 hours. As determined by CBA analysis, Gal-4 potently reduced TNF-α, IL-6, -8 and -10 secretion (p<0.01) (Fig. 4A). Using the same experimental design as above, we transfected PBT with specific Gal-4 antisense to investigate the role of endogenous Gal-4. Gal-4 expression was inhibited by 49±7%. Interestingly, in contrast to the lack of Gal-4 antisense to interfere with the T cell cycle, blockade of Gal-4 expression increased TNF-α secretion of activated T cells by 86.0±9% and IL-6 secretion by 23.0±3%. Next, we investigated the effect of Gal-4 on the cytokine secretion profile of CD2-stimulated LPT. Comparable to PBT, Gal-4 reduced the secretion of TNF-α by 18±3%, IL-6 by 22±1.5%, IL-8 by 11±2.8% and IL-10 by 7±2%, respectively. Furthermore, when PBT were activated by anti-CD3 and LPT by anti-CD2 and cultured for 48 h IL-17 secretion, determined by an IL-17 specific ELISA, dropped by 20±2.5% and 29±3%, respectively (Fig. 4B). We further performed a TNF-α secretion assay based only on living cells. T cells were stimulated for 48 h with anti-CD3/CD28 mAb in the presence or absence of 100 µg/ml Gal-4. After restimulation with PMA (phorbol 12-myristate 13-acetate) and ionomycin, the secreted TNF-α was bound to the surface of the secreting cells, stained and detected by flow cytometry. In addition, T cells were stained with PI to exclude dead cells for further analysis. The respective results (not shown) were comparable to the results depicted in figure 4.

Having described the cell-binding capacity of Gal-4 and its ability to modulate T cell activation, cycling and expansion as well as cytokine secretion, we next addressed the question of whether its cell binding induces apoptosis.

### Induction of T cell apoptosis by Gal-4

**Figure 5:**
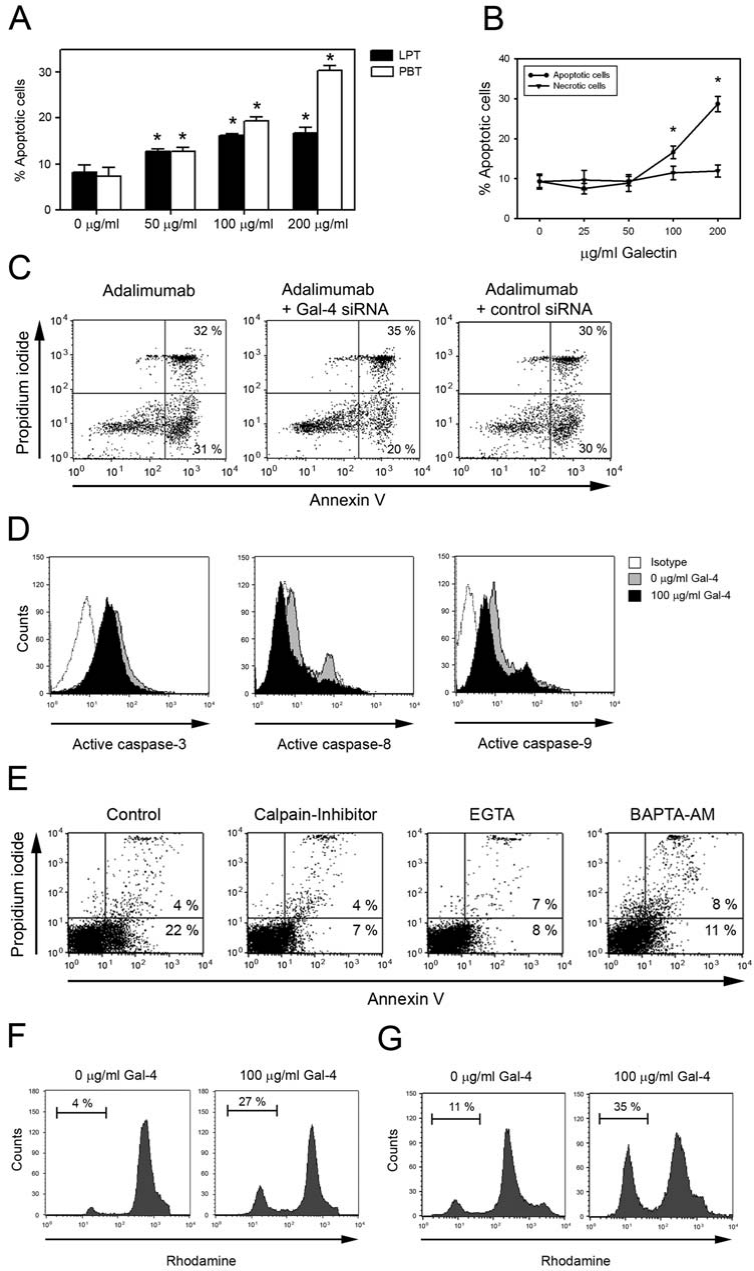
Galectin-4 induces T cell apoptosis caspase-independently via calpain-mediated pathways. (A) PBT and LPT were stimulated with anti-CD3/CD28 or -CD2, respectively, in the presence of 0, 50, 100 or 200 µg/ml Gal-4 and apoptosis was determined by annexin-V staining. Data represent mean±SEM of eight individual experiments. *p≤0.05 for increase vs. baseline. (B) PBT were stimulated in the presence of 0, 25, 50, 100 or 200 µg/ml Gal-4. Apoptosis was detected by annexin V/PI staining, necrosis by PI staining followed by flow cytometry. Data represent mean±SEM of six individual experiments. *p≤0.05 for increase vs. baseline. (C) PBT were transfected with Gal-4 siRNA or scrambled control, activated by anti-CD3/CD28 and cultured in the presence of the TNF-α blocker adalimumab for three days. Apoptosis was detected by annexin V/PI staining followed by flow cytometry. Data are representative for three individual experiments. (D) Caspase-3, -8, and -9 activity in anti-CD3/CD28 stimulated T cells cultured in the presence or absence of 100 µg/ml Gal-4. Data are representative for three individual experiments. (E) PBT were activated by anti-CD3/CD28 and cultured for 24 h in the presence or absence of 100 µg/ml Gal-4 and 50 mM Calpain inhibitor z-LLY-fmk, 4 mM EGTA or 30 mM BAPTA-AM. Data are representative for three individual experiments. (F) Disruption of the mitochondrial membrane potential was detected by rhodamine123 staining followed by flow cytometric analysis. T cells were stimulated with anti-CD3/CD28 and incubated for 3 h in the presence or absence of 100 µg/ml Gal-4. Afterwards cells were analysed by flow cytometry. All data are representative for three individual experiments. (G) Disruption of the mitochondrial membrane potential was detected after 12 h by rhodamine123 staining followed by flow cytometric analysis. All data are representative for three individual experiments.

Using the externalization of phosphatidylserine as a marker of cell apoptosis and positive DNA staining as an indication of membrane leakage, we tested the ability of Gal-4 to induce PBT and LPT cell death. As depicted in figure 5A, Gal-4 induced dose-dependently apoptosis of activated PBT and LPT, but not resting T cells. In contrast to its strong induction of T cell apoptosis, T cell necrosis, as verified by PI staining, was not induced by Gal-4 (Fig. 5B). Having verified the strong capacity of Gal-4 to induce T cell death, we next tested if endogenous Gal-4 might be also involved in apoptosis regulation. Indeed, blockade of Gal-4 expression by specific antisense oligonucleotides modestly reduced adalimumab-induced T cell death from 63% of cells to 53% (Fig 5C).

Since 50 mM lactose was effective in impairing binding of Gal-4 to T cells, we reasoned that cell viability should remain unaffected when blocking carbohydrate-dependent binding. This was indeed the case in Gal-4, while sucrose presence proved inert (not shown). Furthermore, treatment with 10 nM and 1 µM cycloheximide did not inhibit Gal-4 induced T cell apoptosis, indicating that abolishment of *de novo* protein synthesis is not required for its proapoptotic activity (not shown). Having excluded the need for protein synthesis to induce T cell death, we next aimed to investigate the pathways by which Gal-4 causes T cell death.

### Gal-4-mediated induction of T-cell apoptosis is caspases-independent but calpain-dependent

Caspases are central initiators and executors of T cell apoptosis and we previously demonstrated that Gal-2 induces T cell death via caspase-3 and -9 signaling [9]. We thus investigated caspases-3, -8 and -9 activity in Gal-4 treated anti-CD3/CD28 stimulated PBT using fluorogenic substrate assays. As depicted in figure 5D, neither the activity of the up-stream caspases-8 or -9, nor the activity of the central executor caspases-3 was induced by 100 µg/ml Gal-4, a dose sufficient to induce T cell apoptosis (Fig. 5B). Accordingly, the pan-caspase inhibitor zVAD-fmk (50 mM) and the caspases-3 inhibitor zDEVD-fmk (50 mM) failed to prevent Gal-4 induced apoptosis of activated T cells (not shown).

Consequently, we went on to investigate caspase-independent apoptotic pathways [14]. Calpains are a family of calcium-dependent thiol-proteases that induce apoptosis caspase-independently by proteolyzing a wide variety of cytoskeletal, membrane-associated and regulatory proteins [15]. Indeed, the irreversible calpain-inhibitor Z-LLY-fmk inhibited Gal-4 induced T cell apoptosis by 60% (Fig. 5E). Aiming to independently confirm the role of calpain signaling, we inhibited extra- and intracellular calcium levels by the Ca^2+^-chelators EGTA and BAPTA-AM. As depicted in figure 5E, both inhibitors were able to sufficiently block Gal-4 induced T cell apoptosis, indicating that a preceding Ca^2+^ mobilization is required for Gal-4 to induce T cell death. Accordingly, and confirming the participation of the mitochondrial signaling pathway in Gal-4 mediated T cell death independently, we could demonstrate by rhodamine123 staining that Gal-4 and strongly reduces the Δψ (Fig. 5F and G). To determine if the reduction of the mitochondrial membrane potential was an initiating event we performed a time kinetic and checked the mitochondrial membrane potential 3, 6, 12, 24 and 48 h after incubation with 100 µg/ml Gal-4. As depicted in figure 5F the mitochondrial membrane potential was already diminished 3 h after incubation with Gal-4 and decreased more 12 h after incubation (Fig 5G). The time kinetic showed a continuous decrease of the mitochondrial membrane potential up to 72±4% of cells 48 h after Gal-4 incubation (not shown).

The results so far demonstrated that Gal-4 potently inhibits T cell activation, pro-inflammatory cytokine secretion, and induces T cell apoptosis. In IBD, T cell activation is increased and apoptosis impaired, making Gal-4 an attractive candidate to limit mucosal inflammation. Thus, we went on to evaluate the effect of Gal-4 in a well-established model of experimental colitis.

### Gal-4 ameliorates DSS-induced colitis and induces apoptosis of mucosal mononuclear cells

**Figure 6:**
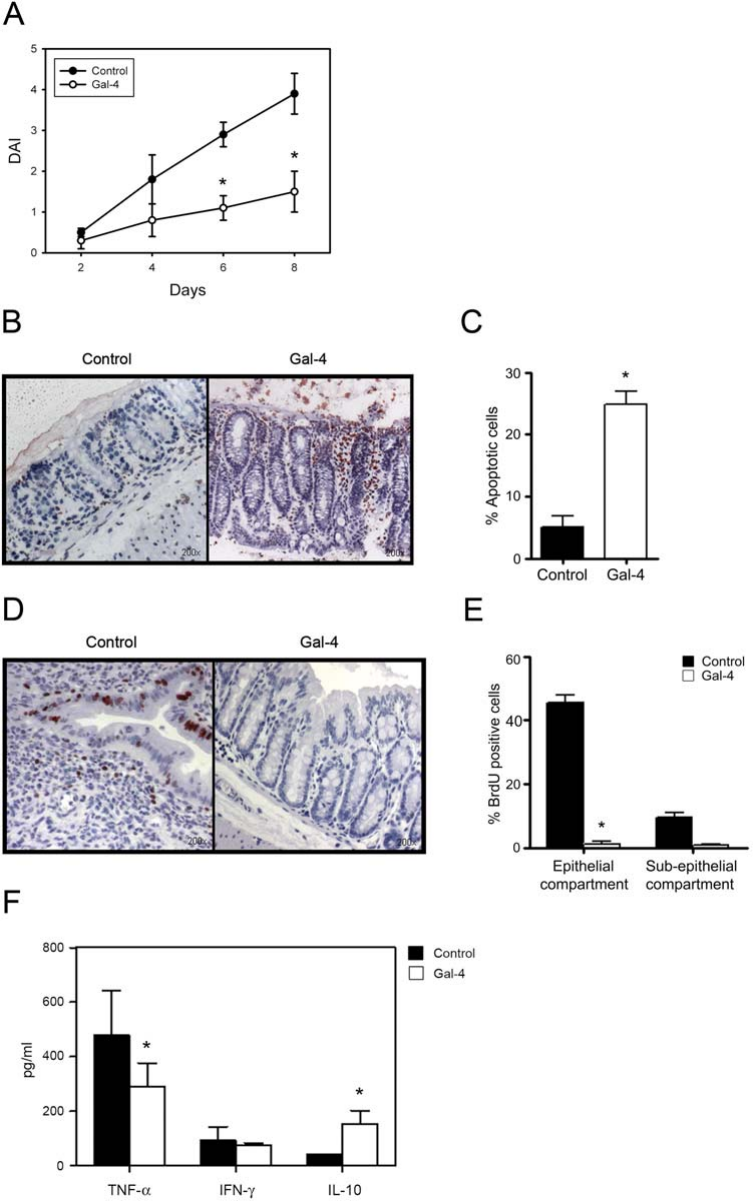
Galectin-4 ameliorates experimental colitis by inducing apoptosis and reduction of pro-inflammatory cytokine secretion. (A) Disease activity index in acute DSS-induced colitis, treated with 0.9% sterile saline (control) or 1 mg/kg BW Gal-4 i.p. three times daily. *p≤0.01 for change vs. control. (B) Detection of apoptotic cells by TUNEL staining in cryosections of colonic tissue from mice with experimental colitis treated with 0.9% sterile saline (control) or 1 mg/kg BW Gal-4 i.p. three times daily. (C) Apoptotic cells after TUNEL staining were counted in three power fields on four slices of different animals by an investigator blinded to the protocol. *p≤0.05 for change vs. control. (D) Detection of proliferating cells by BrdU staining in cryosections of colonic tissue from mice with experimental colitis treated with 0.9% sterile saline (control) or 1 mg/kg BW Gal-4 i.p. three times daily. (E) BrdU positive cells were counted in three power fields on four slices of different animals by an investigator blinded to the protocol. *p≤0.01 for change vs. control. (F) Cytokine secretion was determined by CBA analysis from colonic cultures from mice with experimental colitis treated with 0.9% sterile saline (control) or 1 mg/kg BW Gal-4 i.p. three times daily. All data represent mean±SEM of ten individual experiments. *p≤0.05 for change vs. control.

Acute colitis was induced in Balb/c mice by administration of 5% DSS in the drinking water. The animals were treated either with sterile saline as controls or 1 mg Gal-4/kg BW for 8 days i.p. three times daily until they were sacrificed on day 8. As evaluated with the well-known Rachmilewitz disease activity index (DAI) [16], [17], mice exposed to 5% DSS and treated with sterile saline developed symptoms of acute colitis with diarrhea first, followed by rectal bleeding and substantial weight loss (not shown). In contrast, when mice with DSS-induced colitis were treated with Gal-4, the DAI dropped significantly from 3.9±0.4 to 1.6±0.3 compared to control animals (p < 0.01) (Fig. 6A). Consistent with the results of body weight and DAI scores histological assessment of colonic tissues using the well-established Dieleman score [18], previously described by Cooper [19], revealed that the colons from Gal-4 treated mice displayed significantly reduced mucosal damage and facilitated mucosal repair compared to control mice (not shown). The distinct modulation of the disease activity index and histological damage was also confirmed by a significant reduction of MPO activity in the colonic mucosa of Gal-4 treated mice compared to control animals (p < 0.05) and increased colon length (not shown). Induction of mucosal T cell apoptosis is a highly efficient therapeutic approach [20] and since we demonstrated that Gal-4 induces T cell apoptosis *in vitro*, we next asked if Gal-4 exerts its therapeutic effect by inducing mucosal apoptosis *in vivo*. By using TUNEL (terminal deoxynucleotidyltransferase-mediated UTP end labeling) staining as a well established method for detecting DNA fragmentation, we could show that Gal-4 treatment robustly increased apoptosis of mucosal mononuclear cells (24.8±2.1%), whereas in control animals only few apoptotic cells were detected (5±1.8%) (Fig. 6B, C). As previously discussed, in IBD unrestricted T cell proliferation results in mucosal inflammation, making it desirable to inhibit T cell cycling in mucosal inflammation. Using *in situ* BrdU labeling, we could demonstrate, that in response to DSS application, BrdU is found within the epithelial, but also sub-epithelial compartment but, congruently with the *in vitro* results, cell expansion in Gal-4 treated animals was clearly inhibited, again confirming the applicability of this colitis model and the transmissibility of the *in vitro* data to the *in vivo* model (Fig 6D, E). During acute inflammation a wide range of cytokines influence cell activation, cell adhesion and recruitment of leukocytes such as monocytes, lymphocytes and neutrophils. Thus, to complete the picture, we measured the ability of Gal-4 to modulate cytokine secretion in this model of intestinal inflammation.

### Gal-4 treatment results in a modulation of cytokine secretion

In parallel to the *in vitro* experiments, we first examined cytokine secretion of PBMC, isolated from control and Gal-4 treated animals after three days of additional activation with anti-CD3 mAb. By using CBA assays, we demonstrated that Gal-4 reduced the secretion of the pro-inflammatory cytokine TNF-α by 21±3.8%, while the release of IFN-γ was not altered (not shown). With regard to anti-inflammatory cytokines, Gal-4 increased IL-10 secretion by 29±2% compared to controls (not shown). Finally, we measured mucosal cell cytokine secretion in our model by using the colon culture system [21]. As depicted in figure 6F, compared to controls, Gal-4 treatment reduced TNF-α secretion by 40±6.4% (p < 0.05), increased IL-10 secretion by 400±9.6% (p < 0.01), while IFN-γ secretion was not changed.

## Discussion

Due to growing scientific evidence, the important role of galectins as central regulators of immune and inflammatory responses is undisputable [5]. Nevertheless, little information is available about the functional role of galectins during mucosal inflammation, where a disturbed innate and adaptive immune system triggers local and systemic inflammation.

Gal-1 is so far the best investigated member of the galectin family and Santucci and coworkers demonstrated that the proto-type galectin Gal-1 suppresses experimental colitis [22]. However, Gal-1 is constitutively expressed in most organs [5], showing that Gal-1 broadly, but also nonspecifically, interact with the immune system. In contrast, the tandem-repeat type Gal-4 is exclusively expressed within the GI tract which suggests that Gal-4 might play a more defined and selective role within the intestinal mucosa, prompting us to investigate the role of Gal-4 in mucosal inflammatory processes.

By performing sequential immunohistochemical analysis, we showed strong Gal-4 expression in epithelial cells along the alimentary tract and thus confirmed the previously described RNA expression profile [7]. Additionally, we demonstrated that Gal-4 is secreted by intestinal epithelial cell lines, a pre-requirement to interact with the underlying cells of the lamina propria. T cell dysfunction is undoubtedly a key feature in the initiation and perpetuation of IBD and by demonstrating that Gal-4 binds to activated peripheral and mucosal T cells, we set ground to further investigate the role of Gal-4 in the adaptive immune system. Interestingly, our finding that Gal-4 binds only to activated, but not resting T cells, might explain the recently described phenomena by Hokama et al., that the reactivity of CD4+ T cells to Gal-4 is only elicited under inflammatory conditions [10]. The selective binding patterns of Gal-4 suggested that Gal-4 binding sides might be associated with surface regions involved in the activation of T cells and in deed, Hokama et al. reported that Gal-4 binds to CD4+ T cells at the immunological synapse [10]. We could now confirm this finding and demonstrated, that Gal-4 homes in on the CD3 region, and thus provide evidence that not only the prototype galectins [8], but also the tandem-repeat galectins associate with this receptor complex.

The CD3 receptor complex is required for cell-surface expression of the antigen-binding chains, T cell signaling and activation. Thus, the binding of extracellular Gal-4 to this complex or a tightly associated glycoprotein as major target implies that Gal-4 is capable of modulating central T cell functions. The strong down-regulation of CD25 expression of CD3-activated T cells by Gal-4 proves the biological consequence of the Gal-4 binding to the CD3 complex. Performing a detailed analysis of essential cell cycle checkpoints, we revealed that the restricted activation of T cells by Gal-4 is carried through the whole cell cycle and finally results in a declined mitosis and expansion of the respective T cell population. Proving the concept that the binding of extracellular Gal-4 is linked to the capacity of Gal-4 to inhibit cell cycling, blockade of endogenous Gal-4, which is not linked to the CD3 complex, failed to modulate T cell cycling.

T cell cycling and cytokine secretion are ultimately linked [13]. Adding evidence to this association, Gal-4 potently reduced TNF-α, IL-6, -8, -10, and -17 secretion. Although TNF-α serum and mucosa levels seem to be variable in IBD [23], [24], it is well recognized that this cytokine plays a crucial role in the pathogenesis of IBD and its blocking is effective in the therapy of IBD [23], [25]. With regard to IL-6, a broad spectrum cytokine with characteristics of an acute-phase reactant, mucosal cytokine levels are elevated consistently in IBD tissues and neutralization of soluble IL-6 receptors *in vivo* causes suppression of colitis activity and induction of apoptosis [26]. Interestingly, it was recently demonstrated by Hokama et al. that Gal-4 increases IL-6 secretion in CD4^+^ T cells isolated from TCR-α knockout mice with a unique subset (TCRα^−^β^+^), but not in CD4^+^ T cells from wild-type mice, confirming the association of Gal-4 to the TCR region and indicating that the ability of CD4^+^ T cells to produce IL-6 in response to Gal-4 is dependent on the presence of TCR mutations [10].

The idea that Gal-4 might restore naturally occurring anti-inflammatory systems which are defective in IBD was substantiated by our observation that Gal-4 potently induces PBT, but also LPT apoptosis. Having said so, it was now crucial to investigate if in reverse blockade of endogenous Gal-4 is capable to reduce T cell death. Uncovering the involvement of Gal-4 in the apoptotic program of T cells and assigning a new biological role to endogenous Gal-4, in deed, inhibition of Gal-4 expression by specific antisense oligonucleotides decreased TNF-α inhibitor induced T cell apoptosis substantially.

The potency of galectins to induce T cell apoptosis is common to the prototype and the chimera type galectins, however, the underlining pathways are distinct. Whereas Gal-1 induces PBMC apoptosis via caspase activation [9], [27], Hahn et al. reported, that T cell death induced by Gal-1 in MOLT-4 is mediated caspase-independently [28]. Recently, Stowell and coworkers reported that human Gal-1, -2, and -4 induce death of neutrophil granulocytes, but failed to induce cell death in the leukemic cell lines MOLT-4 and HL-6 [29]. However, both cells lines have disabled apoptotic programs [30], incongruous to directly compare the results. Furthermore, however speculative, the deletion within the TCR-α locus of MOLT-4 [31], might contribute to the described resistance of MOLT-4 towards Gal-4 induced cell death. Although Gal-4 induces T cell death carbohydrate-dependent without need of *de novo* protein synthesis, a feature shared with Gal-1, Gal-2 and Gal-9 [9], [15], [28], Gal-4 neither activated caspase activity nor was its pro-apoptotic effect blocked by caspase inhibitors. Our study now provided further evidence that galectins distinctively induce T cell apoptosis and that both, caspase-dependent and caspase-independent death pathways can be initiated by galectins. In deed, we identified calcium-calpain signaling as a crucial pathway by which Gal-4 induces T cell death. This trail is shared with the tandem-repeat-type galectin-9, but not Gal-2 [9], adding to the evidence that the galectins investigated so far, despite the common carbohydrate binding to T cells, all induce T cell death distinctively.

In IBD, T cell activation is increased and apoptosis impaired, making Gal-4 an attractive candidate to potentially limit mucosal inflammation. Thus, we went on and evaluated the effect of Gal-4 in a well-established model of experimental colitis [32]. When colitis was induced in mice by DSS application, Gal-4 treatment significantly ameliorated clinical signs of inflammation, as well as histopathological signs of mucosal inflammation. The corresponding decrease in neutrophil infiltration and reduced MPO activity in the intestinal mucosa further confirmed the biological relevance of this finding. Despite current discussion, it is well accepted that reasoned by the fact that mucosal T cell apoptosis is impaired in IBD [33], the induction of mucosal T cell death is a highly efficient approach in the treatment of both CD and UC [20]. Thus, the induction of apoptosis of mucosal cells by Gal-4 not only translates the *in vitro* results to the *in vivo* situation, but also explains the therapeutic effect of Gal-4 in experimental colitis. Mucosal inflammation is initiated and perpetuated by the secretion of pro-inflammatory and chemotactic cytokines and potent anti-inflammatory drugs are able to inhibit their release [34], [35]. Thus, the potent down-regulation of peripheral and also mucosal TNF-α and IL-10 secretion by Gal-4 underscores the potential therapeutic use of Gal-4 and explains its beneficial effect in experimental colitis.

Our results systematically and sequentially uncovered the expression and secretion profile of Gal-4 as well as a previously unrecognized biological function of this lectin. We could demonstrate that Gal-4 potently binds to activated T cells at the CD3 region which is translated in a potent inhibition of cell cycle progression and induction of antigen-induced cell death. By ameliorating experimental colitis via induction of mucosal T cell apoptosis and the potent inhibition of pro-inflammatory cytokine release, we identified gastrointestinal Gal-4 as a potential novel candidate to treat mucosal inflammation.

## Materials and Methods

### Isolation and culture of T cells

PBMC were isolated from peripheral blood samples, lamina propria T cells (LPMC) from surgical specimen, essentially as previously described [9], [36]. Signed informed consent was obtained from each subject. Approval of the protocol and consent form was granted by the local ethics committee of the Charité (Berlin, Germany). For isolation of T cells specific magnetic cell sorting (Miltenyi Biotech) was performed negatively selecting the CD3^+^ population. Cells were cultured in RPMI 1640, containing 10% fetal calf serum, 1.5% HEPES buffer, 2.5% penicillin-streptomycin and stimulated with plate-bound anti-CD3 mAb (clone OKT3; kindly provided by Janssen-Cilag, Neuss, Germany) and anti-CD28 mAb (ANC28.1/5D10; Ancell Corp.), LPT with anti-CD2 (T11_2_ and T11_3_; generously provided by Ellis Reinherz, Dana Farber Cancer Institute, Boston, MA). T cells were cultured for 24, 48 or 72 h in the presence or absence of 100 µg/ml Gal-4 (R&D Systems).

### Immunohistochemistry for Gal-4 expression

Immunohistochemical staining was performed using biopsies from human colonic tissue of healthy volunteers. Patients underwent colonoscopy for gastrointestinal symptoms such as abdominal pain or changed stool habits. Histological examination of the control subjects showed no inflammation. Signed informed consent was obtained from each subject. Approval of the protocol and consent form was granted by the local ethics committee of the Charité (Berlin, Germany). Expression of Gal-4 was revealed using a goat anti-human Gal-4 polyclonal Ab (R&D Systems) followed by a biotinylated anti-goat-Ab (Vector laboratories). Negative controls were incubated with the biotinylated anti-goat-Ab only. Complexes were uncovered by adding avidin-biotinylated enzyme complex to all sections in the presence of 3-amino-9-ethylcarbazole (AEC) (Vector laboratories). Nuclear staining with hematoxylin (HE) was performed subsequently. The slides were studied in Axioskop (Zeiss) digital photographs were taken with Axiocam (Zeiss).

### Galectin-4 secretion

Levels of Gal-4 were determined in supernatants of HT-29 and Caco-2 cells cultured for 48 h. Gal-4 was detected by ELISA using an anti-human Galectin-4 capture Ab (R&D Systems) and a biotinylated anti-human Galectin-4 Ab (R&D Systems) and visualized by streptavidin-horseradish peroxidase and TMB substrate (both from BD Pharmingen).

### Galectin-4 binding to T cells

Determination of Gal-4 binding to T cells was performed by incubating resting and for 48 h CD3/CD28-stimulated PBT and CD2-stimulated LPT with 5 µg/ml Gal-4, a dose not able to induce T cell apoptosis. Binding was detected 24 h after incubating cells with Gal-4 by a goat anti-human Gal-4 polyclonal Ab followed by staining with PE-labeled secondary Ab (BD Pharmingen) followed by flow cytometric analysis. Carbohydrate-dependence of the binding was analysed by addition of 50 mM lactose as a pan-galectin inhibitor and 50 mM sucrose as control.

### Polymerase chain reaction

Total RNA was isolated using RNA Bee RNA isolation solvent (Tel-Test Inc.). 2.5 µg of total RNA were reverse transcribed and Gal-4-specific mRNA was amplified using the following primers: sense 5′-TGGTAAATGGAAATCCCTTCTATG-3′, antisense 5′-GAGCTGTGAGCCCTCCTT-3′ (Tib Molbiol, Berlin, Germany). The thermal commenced with a hot start at 94°C for 4 min, followed by 40 cycles each consisting of 94°C for 45 s, annealing for 45 s at 58°C, and extension at 72°C for 60 s, and terminated after a final 10-min period at 72°C. The products were separated on a 1.5% Tris-acetate/EDTA agarose gel and visualized by ethidium bromide staining under UV light.

### Transfection of siRNA

For transfection, 5×10^6^ PBMC were resuspended in an optimized transfection solution for primary human T cells (Amaxa). Each sample was transfected with between 0.5 and 10 µM siRNA, according to the optimized protocol for unstimulated human T cells (Amaxa). For Gal-4 silencing a siRNA target pool was used (Dharmacon RNA Technologies), the scrambled control was a non-targeting pool (Dharmacon RNA Technologies). 12 hours after transfection cells were stimulated with IL-2 and cultured in the presence or absence of 100 µg/ml Gal-4 with and without 50 µg/ml adalimumab. Cells were harvested and analysed by flow cytometry 48 h post-transfection. Following isolation of total RNA with RNA Bee, the efficacy of the specific siRNA to downregulate Gal-4 mRNA expression was determined by a TaqMan real-time PCR on a MxPro 3000 (Stratagene, CA) and quantified with Sybr®Green (Applied Biosystems). GAPDH was used to normalize total RNA.

### Magnetic separation of Gal-4-containing complexes

For separation of Gal-4-associated complexes, tosyl-activated supermagnetic polystyrene beads (Dynalbeads; Dynal Biotech) were coated with BSA, Gal-1 (R&D Systems) and Gal-4. After incubation, the beads were washed, blocked in 0.2 M Tris buffer (pH 8.5 containing 0.1% BSA) and incubated with PBT. Free cells were removed by washing, and cells bound to the beads were treated with lysis buffer (20 mM Tris-HCl, pH 7.6, 10 mM MgCl2, 0.05% Triton X-100, and 50 mM phosphatase and protease inhibitor mixtures). The extract was subjected to SDS-PAGE electrophoresis.

### Western blotting

Cells were lysed in cell lysis buffer (1% Triton X-100, 0.5% Nonidet P-40, 0.1% SDS, 0.5% sodium deoxycholate, 5 mM EDTA, 50 mM protease and phosphatase inhibitor mixtures, 1 mM PMSF, 100 µg/ml trypsin-chymotrypsin inhibitor, and 100 µg/ml chymostatin in PBS). 10 µg of each sample were fractionated on a 4-12% tris-glycine gel and transferred to a 0.2 µm nitrocellulose membrane (Invitrogen Life Technologies). The primary and the appropriate HRP-conjugated secondary Ab were purchased from Santa Cruz Biotechnology.

### Flow cytometric analyses

T cells were analysed by flow cytometry as previously described [9] using a flow cytometer (FACSCalibur; Beckman Coulter). To perform analysis of intracellular proteins, cells were fixed in paraformaldehyde and permeabilized by PBS containing 0.5% saponin (Sigma-Aldrich). Cyclin A-PE, Cyclin B1-PE, PE-labeled anti-active caspase 3, CD3-PE, CD4-PE, CD4-FITC, CD25-FITC, CD80-PE, CD86-PE and annexin V FITC were purchased from BD Pharmingen, PI from Calbiochem.

### Analysis of T cell cycling and division

PBMC were activated with plate-bound CD3 mAb and incubated in the presence or absence of 100 µg/ml Gal-4 for 72 h. Staining for DNA content and cyclin B1 follwed by flow cytometry analysis was performed, as described previously [37]. Dead cells were excluded for further cell cycle analysis by proper gating on cells with diploid DNA content. Analysis of cell kinetics with BrdU staining was performed as previously described [37]. In brief, cells were incubated with 25 µg/ml BrdU chased with thymidine. BrdU labeled DNA was stained with anti-BrdU (BD Pharmingen) and PI following denaturation of DNA by acid treatment and analysed by flow cytometry. Analysis of cell division by dye dilution was performed using the Vybrant CFDA SE Cell Tracer Kit (Molecular Probes). Cells were resuspended in PBS with 5 µM carboxyfluorescein diacetate succinimidyl ester (CFDA SE) per 10^6^ cells. After staining, the cells were cultured alone (unstimulated) or in the presence of plate-bound anti-CD3 mAb, CD28 and IL-2 (20 U/ml). After 4 days, the cells were analysed by flow cytometry.

### Analysis of cytokine secretion

To determine cytokine secretion, human PBT were activated by anti-CD3 and anti-CD28 mAb, murine PBMC were activated by anti-murine CD3 (clone 145-2C11; BD Pharmingen; 10 µg/ml) mAbs and cultured for 72 h. For analysis of mucosal cell cytokine secretion pieces of 1 cm^2^ size were resected from the colon of mice and cultured in RPMI complete medium for 24 h. The supernatants were collected and cytokine testing was performed by CBA analysis (BD Pharmingen) as previously described [38]. The secretion of IL-17 by PBT and LPT was determined using an IL-17A specific ELISA (eBioscience).

Further analysis of cytokine secretion of living T cells was performed using a TNF-α secretion assay (Miltenyi Biotec). Human PBT were stimulated with anti-CD3/CD28 mAb in the presence or absence of 100 µg/ml Gal-4. After 72 h cells were restimulated for 1 h with PMA (10 ng/ml) and ionomycin (1 µg/ml) (both from Sigma Aldrich) and the secreted TNF-α was bound to the surface of the secreting cell, stained and detected by flow cytometry. Additionally, T cells were stained with 1 µg/ml PI and cells being positive for PI were excluded for further analysis.

### Measurement of caspase activity

Measurement of caspase activity was previously described [9]. Briefly, PBT were incubated with the PE-labeled anti-caspase-3 Ab, the carboxyfluorescein-labeled caspase-8 inhibitor FAM-LETD-fmk or caspase-9 inhibitor FAM-LEHD-fmk (Biocarta), all binding irreversibly to activated caspases-3, -8 or -9. Cells were analysed by flow cytometry and the increase of caspase activity was determined in comparison to untreated cells.

### Analysis of apoptosis

PBT and LPT were stimulated with anti-CD3/CD28 or -CD2, respectively, in the presence of 0, 25, 50, 100 or 200 µg/ml Gal-4. After 24 h of incubation with Gal-4 apoptosis was determined by annexin-V staining, necrosis by PI staining. For investigation of the apoptotic pathway T cells were stimulated with anti-CD3/CD28 mAb and pre-incubated for 30 min with or without the irreversible calpain-inhibitor Z-LLY-fmk (50 mM) (BioVision), the extracellular Ca^2+^ chelator EGTA (4 mM) and the intracellular Ca^2+^ chelator BAPTA-AM (30 µM) (both from Dojindo, Japan). Afterwards, cells were cultured for 24 h in the presence or absence of 100 µg/ml Gal-4 and apoptosis was detected by staining cells with annexin V/PI followed by flow cytomeric analysis.

### Assessment of mitochondrial membrane potential

Rhodamine123 staining was performed to measure the mitochondrial membrane potential of PBT as previously described [9]. After 3, 6, 12, 24, and 48 h fluorescence analysis was performed by flow cytometry.

### Animals

Female BALB/c mice, weighing 18-20 g, were purchased from Charles River Laboratories (Wilmington, MA). All animals were held in standard caging conditions and received standard rodent chow and drinking water *ad libitum*. All animal experiments were approved by the local institutional review board.

### Treatment protocol

The animals received 5% DSS added to drinking water to induce an acute colitis. The control group was treated with sterile isotonic saline i.p., the second group received 1 mg/kg BW Gal-4 i.p. three times a day. The treatment started with the giving of the DSS drinking water. The animals were weighted daily and sacrificed under anaesthesia (Rompun; Bayer, Leverkusen, Germany and Ketavet; Pharmacia GmbH, Erlangen, Germany) after 8 days of treatment. Blood samples were collected from the heart. The colon was removed and measured followed by further analysis.

### Determination of disease activity and histological grading of colitis

A disease activity index (DAI), previously described by Rachmilewitz [39], was determined by an investigator blinded to the protocol according to the standard scoring system. A fecal occult blood test (hemoCARE®, care diagnostica, Moellersdorf, Austria) was used for haemocult screening. Colonic tissues were removed for histological analysis and MPO activity by division of the colon along. For histological grading the tissues were embedded into paraffin and HE-stained. The sections were scored blindly for histological evidence of inflammation with a scoring system previously described. MPO activity was assessed as previously described [18].

### Detection of apoptosis

Apoptotic cells within the mucosa were detected by enzymatic in situ labelling of DNA strand breaks using the TUNEL reaction. TUNEL staining was performed on cryosections of colonic tissue using the Apoptosis Detection Kit (R&D Systems). Apoptotic cells were counted in three power fields on four slices of different animals by an investigator blinded to the protocol.

### Detection of proliferation

Proliferating cells in the colon were detected by injecting BrdU (BD Pharmingen) i.p. 24 h before section. To visualize the proliferating cells paraffin slides were incubated with biotinylated anti-BrdU Ab followed by strepavidin-HRP and 3.3′-Diaminobenzidine as substrate. BrdU positive cells were counted in three power fields on four slices of different animals by an investigator blinded to the protocol.

### Statistical analysis

Data are expressed as means±the standard deviation of the means. Statistical analysis for significant differences was performed by using analysis of variance, the Student's t test for parametric samples (GraphPad Prism, San Diego, CA).
